# Identifying local barriers to access to healthcare services in Chile using a communitarian approach

**DOI:** 10.1111/hex.13371

**Published:** 2021-10-08

**Authors:** Alicia Núñez, Carlos A. Manzano

**Affiliations:** ^1^ Department of Management Control and Information System University of Chile Santiago Chile; ^2^ Department of Chemistry University of Chile Santiago Chile; ^3^ School of Public Health San Diego State University San Diego California USA

**Keywords:** Chile, community participation, equity, healthcare access, survey

## Abstract

**Introduction:**

Previous research has used proxy variables or a unique construct to quantify healthcare access. However, there is a need for a different model that can handle this multivariable problem. This study seeks to develop a way to measure access to the local healthcare system with higher local resolution.

**Methods:**

A new survey was developed based on communitarian claims, following a behavioural model and an ontological framework. The survey was used to identify local barriers to healthcare services and the local preferences for priority settings. The results were analysed using multiattribute utility functions and individual weights were assigned by a panel of experts. National and regional indexes of access to healthcare were developed.

**Results:**

The survey contained seven modules and 104 questions. It was conducted on 1885 participants at 42 rural and 231 urban locations in three regions of Chile. The total disutility of the identified barriers to healthcare access at the national level was low (0.1448; values ranged between 0 and 1, with 1 representing a higher barrier) and was higher in the northern region (0.1467). The barriers associated with the health‐policy component showed the highest disutility value, and specific barriers for each community were identified.

**Conclusions:**

These results have the potential to improve health decision‐making in Chile and can be used to assess the impacts of new health policy reforms. Although this model was tested in Chile, it can be adapted for use in any other country.

**Patient or Public Contribution:**

Participants contributed to this study by completing a survey, participating in general talks and receiving brochures with the results obtained from this study.

## 1 INTRODUCTION

A definition of a good access to healthcare can be derived from the deep‐rooted concept of ‘good medical attention’: medicine is only good when it is available to everyone who needs it.^1^ A good access to healthcare services has been described as an effective entrance into the health system, including the transportation to the healthcare facility and the total waiting time,^2^ for the first and subsequent visits to complete the required medical treatment.^3^ Therefore, healthcare services utilization has been commonly used as a proxy to measure healthcare access.^4^ However, this has generated confusion between access and the reception of healthcare services, and therefore, some authors have suggested that access should be defined in terms of opportunities, even if those services were not used.^5^, ^6^ Thus, healthcare access can be better defined as the opportunity to seek appropriate medical attention when it is required.^4^, ^7^

Healthcare access is a universal human right and thus represents a permanent concern for local authorities and governments worldwide.^4^ It is guaranteed by the Chilean constitution,^8^ and the government of Chile introduced a series of reforms to the national health system starting in the 1990s with the objective of reducing the existing inequities in healthcare access and coverage. These included special programmes targeting low‐income populations and covering high‐prevalence and high‐mortality diseases.^9^, ^10^ Additionally, several social determinants in health such as socioeconomic level, ethnicity, gender, unemployment conditions and immigration status were targeted as part of the Millennium Development Goals.^11^, ^12^ As a result, the basic health indexes show that the overall quality of the Chilean national healthcare system has been constantly improving in the last few decades.^13^ However, people's satisfaction with the national healthcare system has remained relatively low (35%) compared to the averages seen in members of the Organization for Economic Cooperation and Development (OECD) (70%).^14^ This low satisfaction can be a product of the presence of several barriers to accessing healthcare services and healthcare providers, in addition to the existing gaps between the public and private sectors participating in the national health system.

Financial, psychological, informational, social, cultural, geographic and temporal factors can affect the way people access and utilize the local healthcare system. According to the model proposed by Aday and Andersen,^2^ these barriers are known to increase the inequality at different healthcare levels, and can arise from five dimensions: health policies, healthcare services characteristics, demographic characteristics, healthcare utilization and users' satisfaction.^15^ The Chilean healthcare system has been described using these individual barriers in previous studies and using the healthcare utilization approach. For example, it is known that Chile has the second highest expenditure on private insurance among the OECD countries; it also has a high burden of out‐of‐pocket payment, especially for the low‐income groups.^16^ There is also a gender and age bias, with women paying higher insurance premiums than men,^17^ and elderly people generally having lower benefits and coverage.^18^ There is a deficit situation when compared to the OECD averages, particularly in human resources (1.7 physicians in Chile vs. 3.2 in the OECD per 1000 people) and infrastructure (2.2 hospital beds in Chile vs. 4.8 in the OECD per 1000 people).^13^, ^16^ The utilization of laboratory exams, medical and dental visits is concentrated on the richest households, while emergency visits were more prevalent among the poorest households,^19^ and the evolution of healthcare utilization in the past 25 years has shown that local population characteristics (i.e., age, ethnicity, religion, income level) are important factors determining the health outcomes.^19^ Unemployment rates have also been shown to be important to describe the difficulties that informal workers face when accessing local health insurance programmes in Chile.^20^

However, these approaches have focused on using healthcare utilization as a proxy to evaluate the access to healthcare and have not evaluated the opportunity of using the healthcare system as experienced by the population. The objective of this study was to develop and apply a representative survey to identify the main healthcare access barriers that people in the north, central and southern regions of Chile face and identify based on their own experiences. The survey was based on an ontological framework reported previously; it was applied to three different population groups identified with different ethnicities using multistage stratified cluster random sampling. The results were analysed using multiattribute utility functions to develop indexes of access for each region independently. The results would be useful in defining the specific barriers that will set the basis to evaluate the relative inaccessibility to the system and will also provide useful information for priority‐setting.

## 2 MATERIALS AND METHODS

### 2.1 Designing the survey

**Figure 1:**
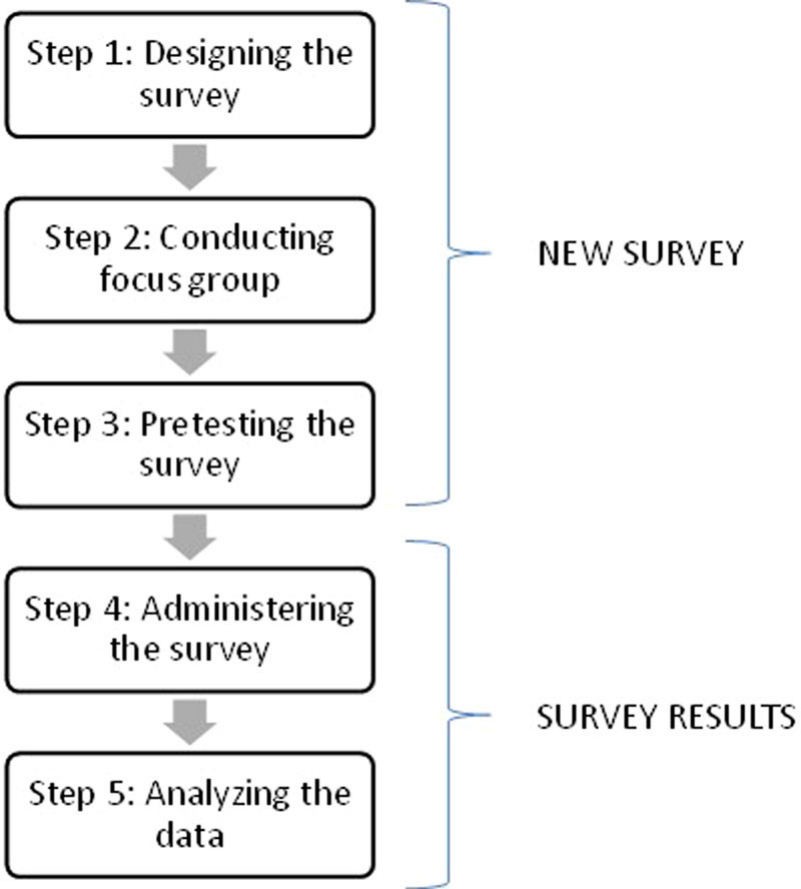
Methodology

The first step in the proposed methodology was to create the new survey (Figure 1). The communitarian claims were the foundation of our definition of equity. Equity is commonly referred to in terms of access and need. Thus, the fundamentals of communitarians about access and equity state that the preferences of the community should determine the principles of any concept of access and how equity is going to be operationalized.^21^ We do believe that individuals are aware of and concerned about the needs and relationships in the society, both within their own groups and between other groups.^22^ Communitarians place the community in the centre of any analysis. Thus, the survey was designed following these principles using a framework based on a modified version of the behavioural model of Aday and Andersen^2^ and using an ontological framework reported previously.^23^ The ontological framework was used to map all the possible elements describing the barriers to access and to validate that all the relevant variables were being incorporated into the final version of the survey. Since all the dimensions from the ontology were included, different constructs derived from healthcare access were considered, thus avoiding problems related to using a unique construct (e.g., utilization) to predict access. The final version of the survey also considered that some of the variables included in the model have short‐ or long‐term viability to alter healthcare access through the development of policies.^24^ For example, we cannot change conditions such as the ethnicity, gender or age of the individuals, but healthcare satisfaction can be modifiable in the short run.

### 2.2 Conducting a focus group

A focus group was used as a qualitative exploration technique to evaluate and validate the survey. This activity lasted approximately 3 h. Seven people participated, four men and three women, who evaluated the designed survey. As a result, some parts of the survey were reformulated to eliminate unclear or misleading questions.

### 2.3 Pretesting the survey

A pilot study was conducted to evaluate the feasibility of conducting the survey and the quality of the questions created. In this context, the individual analysis of the answers from each respondent was the main variable analysed. In addition, comments made by the interviewers, both during the training session and during the pilot study, were incorporated to improve the overall quality of the survey and to establish the final version to be applied in the field. The pilot study was carried out between 8 and 13 February 2017, and the coverage was limited to five randomly selected districts within the Metropolitan Region (RM) of central Chile, including 60 home addresses, and obtaining a total of 30 interviews. Each interviewer was given a set of registration sheets, a tablet, informed consent forms and survey forms. No major problems were reported regarding the general wording and comprehension of the questionnaire. However, we detected the following issues: (a) due to problems of access to the originally selected households located in condominiums or apartments, we had to write a cover letter addressed to administrators and building custodians. (b) If there were three or more households in a dwelling, we decided that the main household was the one to be interviewed. (c) There were people with more than one chronic disease, and thus we modified the survey to collect additional information about other diseases. (d) Not all people had knowledge about the amount of reimbursement received. Therefore, it was necessary to add a new code: ‘99. Don't know’ in questions G3 and G4. We also added a ‘Don't know’ alternative to question F4. (e) Question skipping bugs in the software were also fixed. (f) The use of a card holder was recommended for some questions (B3, B4 and D5). (f) The statement of the alternatives for questions E1 to E8 was rewritten.

### 2.4 Administering the survey

The final version of the survey was administered in regions of the north (Region II), south (Region VII) and central (RM) Chile, which were selected conveniently, given their number of ethnic groups and population size. The sample frame was based on the 2002 National Population and Housing Census.

The method applied to this study can be defined, in addition to being random, as stratified, cluster, multistage and with an application of the systematic simple random method in the selection of the units to be surveyed. To incorporate indigenous communities into the analysis, it was decided to consider the geographical area as a stratum, since the rural area has a greater proportion of indigenous communities. Therefore, a stratified sampling was used according to the conjunction of the Region and Geographic area to which the individual belongs. The objective of this stratification was to apply different sampling strategies in urban and rural areas, in accordance with the different forms of grouping of the population, to obtain more precise estimates and with similar statistical errors in each stratum, which allowed us to compare among results. Clusters were used to improve the quality of the data collected and to facilitate the identification of households to be surveyed while reducing travel times and costs for the interviewers. A systematic simple random sampling allowed each household to have the same probability of being selected, and therefore achieving a better dispersion of the sample. Once the composition of the home was defined, we selected a person from all the individuals, present or absent at the time of the visit, using a Kish Table. In the case of children under 15 years old, the person in charge of the family group was asked to respond to the survey.

Our sampling units were as follows:

(1) Primary Sampling Units included urban and rural areas. In the urban stratum, all communes with a population larger than 100,000 had the same probability of selection, while those communes with a population below 100,000 had a probability of selection proportional to their population. Regional capitals were always included. In the rural stratum, all communes had a probability of selection proportional to their population.
(2) Secondary Sampling Units included the census blocks (conglomerates of dwellings defined by the Census). As many blocks or clusters of five dwellings were required to complete the sample in each stratum.
(3) Tertiary Sampling Units included the private dwellings permanently occupied at the time of updating the sampling frame. In each block, five private homes were selected with equal probability.
(4) Ultimate Sampling Units included the household members, who were randomly selected by the same interviewer at the time of the first visit using a Kish Table.

The survey was conducted between 22 March and 31 May 2017 and included a total of 1885 participants. If the selected person was not contacted or refused to respond after three attempts, the replacement household was chosen using a pendulum system. Tablets and notebooks were used to record the responses, and helped in the validation (i.e., identifying inconsistent responses) and confirmation processes (i.e., through cross‐questioning). The fieldwork team included a project coordinator, three regional coordinators and 33 interviewers. Interviewers were trained and monitored in the field.

We measured the internal consistency of the questionnaire using a Cronbach's *α* test, resulting in coefficients above .6 for each dimension, which is an acceptable value. The results therefore showed high inter‐item correlation and an acceptable level of internal consistency. Principal component analysis was used to determine the validity of each item and identify the underlying components.

### 2.5 Analysis of the data

The multiattribute utility theory was used to assess the disutility produced by the barriers to access to healthcare services, based on the participants' responses. The barriers to healthcare access were defined according to a series of attributes (see Table 1 for an example). Each attribute was subdivided into several levels, such that each participant could be classified into one level for each attribute. In the example from Table 1, the different levels represented the access to medical attention from a specialist, going from greater access (when people received care from a specialist), and decreasing access if the person received care from a general practitioner, a nurse, a healthcare professional and reaching less access (when people did not receive care when it was needed). In this study, we use the multiplicative form of a multiattribute utility function (Equations 1 and 2) (over the linear‐additive and multilinear forms), given that the number of attributes was at least 5.^25^

(1) $$u\left(x\right)=\left(\frac{1}{k}\right)\left[\prod_{j=1}^{n}\left(1+kk_{j}u_{j}\left(x_{j}\right)\right)-1\right],$$

(2) $$\left(1+k\right)=\prod_{j=1}^{n}\left(1+kk_{j}\right),$$

**Table 1:** Example of the barrier classification system to the question: ‘From the point of view of your need for health care attention from a medical specialist, which of the following options best represents you?’

Level	Cod	Description
1	H1	I received medical care from a specialist
2	H2	I received medical care from a general practitioner
3	H3	I received medical care from a nurse
4	H4	I received medical care from other professionals of the healthcare area
5	H5	I received medical care from other persons, not professionals, from the healthcare area
6	H6	I did not receive medical care due to lack of specialists
7	H7	I did not need the medical attention of a specialist


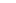
where *u(x)* is the multiattribute utility function for each individual, *k*_*j*_ represents the weight attached to the attribute *j* (i.e., the importance of this barrier in determining the utility score of overall access to healthcare) and the parameter *k* represents the interaction in preferences among attributes. Since we care about the disutility that these barriers can produce in the access to healthcare, an alternative to Equation (1) is to measure the multiplicative multiattribute for the disutility function as follows (Equations (3), (4), (5)):

(3) $$\bar{u}\left(x\right)=\left(\frac{1}{c}\right)\left[\prod_{j=1}^{n}\left(1+cc_{j}\bar{u}\left(x_{j}\right)\right)-1\right],$$

(4) $$\left(1+c\right)=\prod_{j=1}^{n}\left(1+cc_{j}\right),$$

(5) $$c_{j}=\bar{u}(x_{j}^{0},x_{j}^{*}),j=1\text{…}n,$$

where *c*_*j*_ can be determined by measuring the disutility of the specified attribute relative to *u*(x*) = 0 (the best level on attribute *j* according to the individual) and *u*(*x* ^0^) = 1 (the worst level on attribute *j* according to the individual). Thus, the estimated function was based on the preference scores that were obtained from our representative random sample of the regions studied.

The assessment of weights for the attributes is also necessary, since disutility levels for any attribute range between 0 and 1, and therefore any attribute would be of equal importance. To compute *u*_*j*_(*x*_*j*_), we used Equation (6):

(6) $$u_{j}\left(x_{j}\right)=1\times \left(1-\left(\frac{x_{\max}-x_{i}}{x_{\max}-x_{\min}}\right)\right).$$

In this study, the values or scores corresponded to the medium‐level response computed by the arithmetic mean of the collected data.

Once the disutility function was established, the parameters for the final value of *u*_*j*_ *(x*_*j*_ *)* were determined. This is usually performed under an arbitrary process of expert judgement, weighting attributes that are theoretically most influential in the final answer (i.e., access barriers to health). The opinion of 12 experts was considered in this study, including researchers and academics from related fields, surgeons from different specialties and clinical managers in both public and private institutions. Their opinion was useful for the prioritization and weighting of attributes according to their importance. We also conducted parallel computational simulations to check the performance of these models. The multiattribute disutility function was computed after each single attribute disutility was obtained and using an appropriate aggregation rule. Table 2 shows this model through a simplified scheme as an example.

**Table 2:** Simplified scheme

Attributes	Items	Score	Disutility	Weight	Weighting (*C_i_*)
Health policy barriers	Financing	${\bar{x}}_{11}$	${\bar{u}}_{11}$	$w_{11}=c_{1}/\#\mathrm{items}$	$c_{1}$
	Education	…	…	…	
	Manpower	…	…	…	
	Organization	${\bar{x}}_{14}$	${\bar{u}}_{14}$	$w_{14}=c_{1}/\#\mathrm{items}$	
Characteristics of the healthcare services barriers	Labour resources	…	…	…	$c_{2}$
	Capital resources	…	…	…	
	Organizational resources	…	…	…	
Barriers to utilization of healthcare	Type	…	…	…	$c_{3}$
	Site	…	…	…	
	Purpose	…	…	…	
	Time interval	…	…	…	
Consumer satisfaction barriers	Convenience	…	…	…	$c_{4}$
	Cost	…	…	…	
	Coordination	…	…	…	
	Courtesy	…	…	…	
	Information	…	…	…	
	Quality	…	…	…	
Characteristics of the population at risk barriers	Predisposing factors	${\bar{x}}_{51}$	${\bar{u}}_{51}$	$w_{51}=c_{5}/\#\mathrm{items}$	$c_{5}$
	Enabling factors	…	…	…	
	Need factors	${\bar{x}}_{53}$	${\bar{u}}_{53}$	$w_{53}=c_{5}/\#\mathrm{items}$	

A Levene test, followed by a post hoc test and ANOVA were used to assess the differences between regions once the initial results were obtained. Data management and statistical analyses were performed using STATA version 13, SPSS and R. SPSS was used for the utility analysis using an in‐house developed code.

We developed a more user‐friendly Excel simulator with the integrated database to present the results of the identified barriers for healthcare access, in which the values of each dimension can be obtained at the national and regional levels. Additionally, it allows filtering by healthcare coverage system, work status, average household income, religion, ethnicity, smoking habits or sports habits. The simulator is available upon request.

#### 2.5.1 Patient and public involvement

This study received ethical approval from the ethic committee of the School of Economics and Business, Universidad de Chile. Patients were not involved in this study. Participants received an informed consent form and details on the purpose, requirements, type of participation, confidentiality of the information and contact information of the responsible researcher. The informed consent included two copies, one for the participants and one for the researcher, which were signed before their participation in the survey. All the information was prepared following the ethical guidelines and the topics described in Law 20,120 and 19,628 of Chile. The associated risks of this study do not exceed the possible discomforts that resemble those experienced in everyday life. The results were disseminated through general talks and brochures that each of the respondents received. No individual or his/her information can be identified.

## 3 RESULTS

### 3.1 Framework

**Figure 2:**
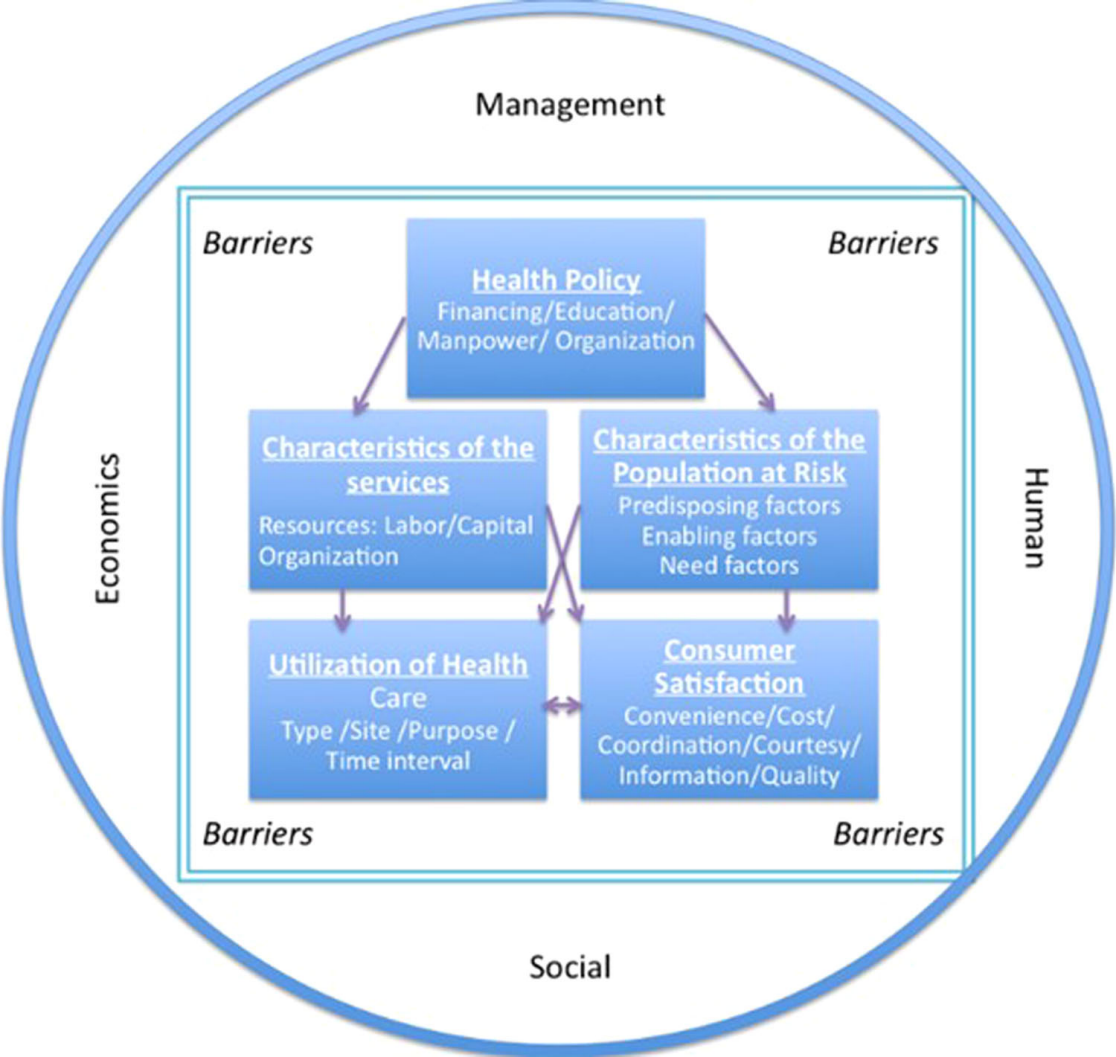
Framework to study healthcare access

The proposed framework (Figure 2) contains the relevant variables to study access to healthcare, which are surrounded by a first layer of undefined barriers, followed by another layer that incorporates four crucial factors to improve the system (i.e., the Management, Economic, Social and Human Infrastructure—MESH Infrastructure) introduced by Mooney and Houston.^26^ If management does not provide adequate support and a good plan to implement the existing policies, the results for the community will not meet the equity requirements. The same idea applies for the economic component, in which it is hard for a community to observe an improvement in their healthcare services if the economic component is inadequate. The social interaction and participation of the community in the decision‐making process are also important factors to measure the improvements in the healthcare services. Finally, the use of human resources must be executed in a proper way to be perceived by the local population. Inadequate infrastructure can further increase the existing barriers. Therefore, MESH plays a dual role: it can help to overcome existing barriers or it can make the system worse if the right actions are not taken.

### 3.2 New survey

The final survey was written in Spanish, although an English version is also available in the Supporting Information, and included seven modules (from A to G) asking open‐ and closed‐ended questions about (A) household composition: 10 questions; (B) personal information of the interviewed: 9 questions; (C) general health information: 11 questions; (D) medical attention: 37 questions; (E) healthcare policies: 13 questions; (F) healthcare services characteristics: 20 questions; and (G) medical expenses: 4 questions. The unit of analysis was the person answering the survey, except in section A (household composition), in which the unit of analysis was each household. The original survey is available upon request.

### 3.3 Survey results

A total of 1885 surveys were collected at 42 rural locations and 231 urban locations (Table 3). Both the disutilities of each attribute and the total disutility of the dimension (calculated as the arithmetic mean of the uni‐attribute disutilities) had associated values ranging between 0 and 1, with 1 representing a higher barrier. The final disutility value was computed weighting each dimension based on the expert judgement. The weights for each dimension were defined according to the median value as follows: characteristics of the healthcare services (37%), utilization of services (21%), satisfaction with the services (12%), characteristics of the population at risk (15%) and healthcare policies (15%). The disutility results based on the use of multiattribute functions are shown in Table 4. The total calculated disutility value of the barriers for healthcare access at the national level was 0.1448; the northern region (II Region) showed the highest score with a value of 0.1467, followed by the southern region (VIII Region: 0.1449) and the central region (RM Region: 0.1427) (Table 4).

**Table 3:** Survey participants

Region	Participants		
	Urban	Rural	Total
II Region (north)	457	168	625
VIII Region (south)	462	168	630
RM Region (central)	462	168	630
Total	1381	504	1885

**Table 4:** Total disutility results and by dimensions of the barriers for healthcare access

National	Score	0.1448		
II Region (north)	Score	0.1467		
VIII Region (south)	Score	0.1449		
RM Region (central)	Score	0.1427		

	National	II Region	VIII Region	RM Region
Health policy	0.841	0.862	0.831	0.830
Characteristics of the healthcare services	0.017	0.017	0.019	0.015
Utilization of healthcare	0.013	0.010	0.015	0.014
Consumer satisfaction	0.025	0.022	0.028	0.026
Characteristics of the population at risk	0.020	0.020	0.021	0.020

The barriers to healthcare access associated with the health policy component showed the highest disutility value at the national and regional levels (>0.83), followed by consumer satisfaction (>0.022), the characteristics of the population at risk (0.020–0.021), the characteristics of the healthcare services (>0.015) and the utilization of healthcare dimensions (>0.010) (Table 4). When analysing the results at the regional level, the population of the northern region (II Region) identified the following major barriers for each dimension as follows:

(1) Health policies: particularly the lack of knowledge of health awareness programmes, especially of prevention and rehabilitation of drugs and alcohol consumption (0.94), and sexual and reproductive health (0.92).
(2) Characteristics of services: the travel time (0.80) and distance (0.20) to healthcare services, lack of availability of different types of healthcare facilities (e.g., rural clinics [0.78], mental health centres [0.61]) and long waiting times to get a medical appointment (0.17).
(3) Utilization of services: the local population identified barriers in the need for medical attention, without receiving it, including specialists and preventive medicine (medical checkups, dental care, ophthalmology) (0.08), and additionally, difficulty in accessing medicines due to lack of availability or high prices (0.05).
(4) Consumer satisfaction: low health insurance coverage (0.18) and unclear language used by the doctor (0.15).
(5) Characteristics of the population at risk: nonexistent or low level of reimbursement in medicines (0.26), homeopathies (0.52) and medical attention (0.21).

The results obtained in the southern and central regions indicated similar barriers to those in the north (see Table 5). In addition, the results of the survey could also be categorized by other groups, such as specific ethnic groups, immigrant population, age groups and many more. An example is shown in Table 6.

**Table 5:** Main barriers to healthcare access by dimension

Region	II Region	VIII Region	RM Region
Variable	Disutility	Disutility	Disutility
Health policy			
Prevention and rehabilitation of drugs and alcohol consumption	0.94	0.92	0.92
Sexual and reproductive health	0.92	0.88	0.90
Characteristics of the healthcare services			
Travel time	0.80	0.77	0.77
Lack of availability of facilities (rural clinics)	0.78	0.67	0.64
Lack of availability of facilities (mental health centres)	0.61	0.67	0.55
Distance to healthcare services	0.20	0.21	0.21
Long waiting times to get a medical appointment	0.17	0.21	0.21
Utilization of healthcare			
Need for medical attention, without receiving it	0.08	0.12	0.10
Difficulty in accessing medicines	0.05	0.09	0.09
Consumer satisfaction			
Low health insurance coverage	0.18	0.18	0.19
Unclear language used by the doctor	0.15	0.21	0.17
Characteristics of the population at risk			
Nonexistent or low level of reimbursement (medicines)	0.26	0.39	0.40
Nonexistent or low level of reimbursement (homeopathies)	0.52	0.31	0.15
Nonexistent or low level of reimbursement (medical attention)	0.21	0.24	0.26

*Note*: The variables shown here (measurable variables) were derived from the generic items shown in Table 2 and are country specific.

**Table 6:** Total disutility results and by dimensions of the barriers for healthcare access among Mapuches, Aymaras & Diaguitas

Filtered by:				
Healthcare coverage	All			
Work status	All			
Average household income	All			
Religion	All			
Ethnicity	Mapuche, Aymara & Diaguita			
Smoking habits	All			
Sports habits	All			
National	Score (2)	0.1447		
II Region	Score (2)	0.1470		
VIII Region	Score (2)	0.1520		
RM Region	Score (2)	0.1378		

	National	II Region	VIII Region	RM Region	
Health policy	0.834	0.841	0.870	0.802	
Characteristics of the healthcare services	0.018	0.021	0.019	0.016	
Utilization of healthcare	0.017	0.017	0.024	0.011	
Consumer satisfaction	0.025	0.024	0.028	0.025	
Characteristics of the population at risk	0.019	0.022	0.017	0.019	

## 4 DISCUSSION

Chile has a complex geographic distribution, which challenges the logistics of good allocation of healthcare resources. Likewise, the characteristics of the people and communities that inhabit each region may differ. Therefore, we cannot expect that everyone will be affected by the same barriers to access to healthcare services. This is the main contribution of the paper; we created a new model for measuring barriers to healthcare access with resolution at the community level. The multiattribute utility theory allowed us to identify which of these barriers to healthcare access are generating the greatest disutility in the local population consulted, and thus identifying the most relevant barriers for each community.

The classification of access to health into the five dimensions of the model (health policy, characteristics of the health service, characteristics of the population, use of health services and user satisfaction) allowed the monitoring of a specific aspect of healthcare services. Therefore, specific actions can be taken, such as the allocation of resources and responsibilities, following a logical model of inputs (health policy) and processes (characteristics of the population and the health service) to achieve results and increase the use of healthcare services and user satisfaction, both being direct evidence of improved access to healthcare.

The overall results of our study revealed that the worst barrier is the lack of information about healthcare awareness programmes among the population, which belongs to the health policy dimension of the model. Most participants ignored health awareness programmes that are available in the country and that have been created to help and prevent major diseases in the population. Other barriers to healthcare access were identified and include the cost of services, excessive centralization of healthcare services in the central region of Chile, lack of specialists and ambulances, shortage of doctors and surgical pavilions, poor infrastructure in some areas, cancellation of medical appointments without previous notice, bad medical attention (‘arrogant, unprofessional, unethical and unempathetic’), poor nighttime attending hours, discrimination between public and private insurance systems and transportation‐related problems (long walking distance, poor connectivity to good public transportation).

From the results of this study, we can say that at the national level, the perceived barriers are similar. Having barriers that are somewhat similar implies that health policies could be promoted at the national level and that they could have a similar impact on people from these three regions. However, this study allowed us to go deeper in our analysis, focusing on different barriers within the same region, but from the point of view of a specific population group. Additionally, within a particular dimension, a region could consider that one specific barrier (e.g., distance to health centres) is greater than the other (e.g., waiting times); therefore, the latter would represent a particular need of this region.

Ignorance of healthcare awareness programme is a barrier that draws a lot of attention, from the point of view of how changing the way messages are transmitted at the government level could generate a measurable impact. On the other hand, the fact that travel times are long for everyone tells us about the logistics in which healthcare centres have been distributed, and that they affect everyone at the national level. Perceiving that there is inequity derived directly from the system is a worrying sign that there are people who required medical services, sought them and still did not receive them. There is also an implicit opportunity having users dissatisfied with their health insurance. Another associated benefit from this study is that the results of the survey could also be categorized by other groups. It does not have to be limited to geographic regions.

This study should be interpreted within the context of several limitations. It includes 3 out of 16 regions of Chile. However, the three regions considered have a high concentration of population, close to 51% of the country's total population. Our subsample did have demographic characteristics like those of the original population. While efforts were made to structure a comprehensive survey, this is a first attempt using this instrument, and would need to expand its application in a much larger sample, including other countries. As such, the findings from this survey are country related. Also, like any survey, it relied on self‐reported data, as individuals' satisfaction, for example, depends not only on the access but also the balance of experience against expectations. We also use expert judgement to compute weights, and like any other type of data, it can be prone to errors and contextual biases. We use structured protocols to ensure that judgements were as reliable as possible.

## 5 CONCLUSION

A new model for measuring the existing barriers to healthcare access was described, based on a communitarian approach, and using an ontological framework, and was applied to identify those barriers generating the greatest disutility in the local population. Once these barriers are identified, specific actions can be taken to increase the use of healthcare services and user satisfaction, both being direct evidence of an improved access to healthcare.

Despite its limitations, which include cross‐sectional and self‐reported data, this study provides information about the main barriers faced by people from three regions of Chile, who voluntarily and randomly gave their opinion. The results revealed that regardless of the complexity of the questions and extension of the survey, along with adequate guidance, the opinion of the Chilean population can be considered for the definition of public policies. In this regard, this study provides valuable information generated by the community and provides a roadmap for improving access to healthcare services. Furthermore, this study is part of a comprehensive research on the access to healthcare and Chilean people's perception in overcoming access barriers to the Chilean healthcare system. We conducted this first survey to identify barriers to access, and then the second survey collected information regarding the communities' preference to overcome the barriers identified. In this way, every community was able to determine its specific barriers that set the basis to evaluate the relative inaccessibility to the system. The second survey developed considers communities' willingness to overcome the barriers detected, considering people's values and preferences for priority, not only thinking about themselves as individuals but also as members of the society and, therefore, considering the entire Chilean population and society.^27^ All these results should contribute to the on‐going debate and research for improving healthcare systems. These results have the potential to improve health decision‐making in Chile and can be used to assess the impacts of the new health policy reforms, understand the barriers faced by the population based on their own opinion and comprehend the changes that are needed to guarantee receipt of all necessary care to all. Policy reforms need to address barriers to access and our results provide this information. Although this model was tested in Chile, it can be adapted for use in any other country in the world.

## CONFLICT OF INTERESTS

The authors declare that there are no conflict of interests.

## ETHICS STATEMENT

Ethical approval has been received from the ethic committee from the School of Economics and Business, Universidad de Chile. Consent was obtained from each participant. No individual or his/her information can be identified.

## AUTHOR CONTRIBUTIONS

Alicia Núñez, principal investigator, developed the original research idea and questions, developed the survey, obtained the data for this study, conducted data analysis, interpreted the results and wrote the manuscript. Carlos Manzano contributed to the interpretation of results, writing and revisions of the manuscript. All authors read and approved the final manuscript.

## Supporting information

Supporting information.

## Data Availability

Data used in this study are available under request from the corresponding author.
