# Supplementary material for: Identifying local barriers to access to healthcare services in Chile using a communitarian approach
Source: Health Expect. 2021 Oct 8;25(1):254–63. doi: 10.1111/hex.13371 (PMC8849372; doi:10.1111/hex.13371)
Supplement: Supplementary file 1 — Supporting information. [file HEX-25-254-s001.doc]

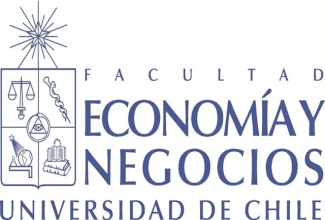
 SURVEY ON ACCESS BARRIERS TO HEALTH

**INTRODUCTION** (read)

Good morning/afternoon: My name is___________, I am part of a survey team working on research conducted by Alicia Núñez Mondaca, academic of the Faculty of Economics and Business from the Universidad de Chile. The objective of this study is focused on identifying barriers of access to the Health Care System in Chile.

The information collected is confidential and its purpose is no other than to know the behavior of people when they access health care services.

Your opinion as a participant is very important for this research. Note that there are no correct or incorrect answers, but different opinions. The relevant issue is to know what really happens when someone access any health service in order to know the possible barriers of the health system. I deeply appreciate in advance for your participation.

1. **General Information**

| Identification of the survey | | | KISH | | Region | | Commune | |
| --- | --- | --- | --- | --- | --- | --- | --- | --- |
|  | | |  | |  | |  | |
| Name | Paternal Surname | | | | | Maternal Surname | | |
|  |  | | | | |  | | |
| Address | | | | Telephone Area code - phone | | | | Mobile |
|  | | | |  | | | |  |
| Village / District | | City | | | | | | |
|  | |  | | | | | | |

| Reserved for Supervision  (In each stage the name of the person responsible must be registered) | | |
| --- | --- | --- |
|  | ID | Name and Surname |
| Name of Surveyor |  |  |
| Name of Supervisor |  |  |
| Name of Encoder |  |  |
| Name of Typesetter |  |  |

**Confidentiality Policy:**

The current research adheres the Organic Law No 17.374 from the National Institute of Statistics, which in its articles states the following: Article 29: “The National Institute of Statistics, Fiscal Organizations, Semi-fiscal Organizations and Government Firms and each of their officers, shall not be allowed to disclose the facts related to determine entities or persons from which it has been brought attention in the course of their activities. The strict maintenance of this reservation constitutes the “STATISTICS SECRET”. The breach by any person subject to this obligation, will incur in committing an offence under the Article 247° of the Criminal Code.

| **A. Household Membership (All the members living at the address)** | | | | | | | | | | | | | |
| --- | --- | --- | --- | --- | --- | --- | --- | --- | --- | --- | --- | --- | --- |
| Including yourself, ¿how many people comprise your household?  *Include all the members of the household.*  *Do not forget minors and elderly people*   | **Total of Persons** | | --- | |  |   Write down all the members of the household,  Start with the household reference person (first) and then the rest of the members | | **A1.** Gender  1. Male  2. Female  3. Transgender  **A2.** Age (actual age)  **A3**. List in descending order all the members of the household and apply the Kish table, according to the letter assigned to the household. SEE CARD  **A4**. Mark the selected person with a letter X, the informant with Y when appropriate  **A5**. What is the current civil status of the members of the household?   1. Married 2. Living with a partner 3. Nulled 4. Separated 5. Divorced 6. Widow 7. Single | | | | | | **A6**. Which is the relationship with the household head?   1. Household head 2. Wife, husband, partner 3. Daughter/son, stepdaughter/stepson 4. Son in law/daughter in law 5. Granddaughter/grandson 6. Sister/brother, sister in law/brother in law 7. Mother/father, mother in law/father in law 8. Other relative 9. No relationship   **A7.** What is the last educational level achieved?   1. Without formal education 2. Preschool/kindergarten 3. Differential Education 4. Primary school incomplete 5. Primary school complete 6. High school incomplete 7. High school complete 8. Technical school incomplete 9. Technical school complete 10. Professional school incomplete (without degree) 11. Professional school complete (with degree) 12. Postgraduate   99. Does not know, no answer | | | **. A8.** Which is your Health Insurance?[[1]](#footnote-2)   1. Fonasa Group A 2. Fonasa Group B 3. Fonasa Group C 4. Fonasa Group D 5. Fonasa (doesn’t know the group) 6. Isapre Más Vida[[2]](#footnote-3) 7. Isapre Colmena 8. Isapre Consalud 9. Isapre Cruz Blanca 10. Isapre BanMédica 11. Isapre Ferrosalud 12. Isapre Vida Tres 13. Isapre (doesn’t know which one) 14. Armed Forces’ Health Insurance[[3]](#footnote-4) 15. Other 16. None 🡪 Go to **A10**   **A9.** Are you Contributor or Beneficiary from your health insurance?   1. Contributor 2. Beneficiary 3. Have an insurance card | | |
| **Order** | **Name** | **A1** | **A2** | **A3** | **A4** | **A5** |  | **A6** | **A7** |  | **A8** | **A9** |  |
| 1 |  |  |  |  |  |  |  |  |  |  |  |  |  |
| 2 |  |  |  |  |  |  |  |  |  |  |  |  |  |
| 3 |  |  |  |  |  |  |  |  |  |  |  |  |  |
| 4 |  |  |  |  |  |  |  |  |  |  |  |  |  |
| 5 |  |  |  |  |  |  |  |  |  |  |  |  |  |
| 6 |  |  |  |  |  |  |  |  |  |  |  |  |  |
| 7 |  |  |  |  |  |  |  |  |  |  |  |  |  |

| **A. Household Membership (All the members living at the address)** |
| --- |

| **A9.** Where would you usually go if you are sick?   1. Hospital 2. Private clinic 3. Public doctor´s office 4. Private doctor´s office 5. Healer 6. Pharmacy 7. Other (specify) | |  | | | | | | | | | |  | |
| --- | --- | --- | --- | --- | --- | --- | --- | --- | --- | --- | --- | --- | --- |
| **A10** | **A10 Specify** |  |  |  |  |  |  |  |  |  |  | |  |
|  |  |  |  |  |  |  |  |  |  |  |  | |  |
|  |  |  |  |  |  |  |  |  |  |  |  | |  |
|  |  |  |  |  |  |  |  |  |  |  |  | |  |
|  |  |  |  |  |  |  |  |  |  |  |  | |  |
|  |  |  |  |  |  |  |  |  |  |  |  | |  |
|  |  |  |  |  |  |  |  |  |  |  |  | |  |
|  |  |  |  |  |  |  |  |  |  |  |  | |  |

| **B. Information of the interviewed (The person selected or interviewed responds about the minor selected)** | | | | | | | | | | | | | |
| --- | --- | --- | --- | --- | --- | --- | --- | --- | --- | --- | --- | --- | --- |
| ***The person SELECTED responds but if minor please refer to as INFORMANT*** | | | | | | | ***About the person SELECTED*** | | | | | | |
| 1. In which of the following situations you spent most of your time last week? 2. Working with a salary 3. Not working, but employed (temporarily absent due to medical license, strike, illness, vacation, temporary suspended or other reason) 4. Working without a salary 5. Looking for a job 6. Working in household tasks 7. Studying **Go to B4** 8. Retired, pensioned or lessor 9. Disable 10. Other situation, specify which one?      1. In your job , You are: 2. Employer 3. Self-employed, independent 4. Private sector wage earners (employee, worker, laborer) 5. Public sector wage earner 6. Housekeeping staff 7. Army or military employee | | | | 1. During the last three months, which was your approximate average liquid income?   *(If you do not perceive remuneration write 0)*   1. Less than $85,741 CLP 2. $85,742 - $228,621 CLP 3. $228,622 - $315,200 CLP 4. $315,201 - $371,054 CLP 5. $371,055 - $470,622 CLP 6. $470,623 - $544,193 CLP 7. $544,194 - $667,302 CLP 8. $667,303 - $878,816 CLP 9. $878,817 - $1,219,273 CLP 10. $1,219,274 - $1,500,000 CLP 11. $1,500,001 - $2,000,000 CLP 12. $2,000,001 - $2,500,000 CLP 13. $2,500,001 - $3,000,000 CLP 14. $3,000,001 - $3,500,000 CLP 15. $3,500,001 CLP or more 16. Does not know / Do not answer 17. Considering the last three months, which was the approximate liquid income of the household?   *Include all incomes perceived by all members of the household, including yourself. For salaries, [[4]](#footnote-5)SUF(Single Family Subsidy), retirement or pension, State Pension, property rental income, among others.*  *(choose a category from B3)* | | | 1. Do you belong to any religion? 2. Catholic 3. Evangelical christian /protestant 4. Other, which? 5. None- Atheist 6. None- Agnostic 7. Do you belong to any ethnic group? 8. Aymará 9. Mapuche 10. Diaguita 11. Other, which? 12. Do not belong 13. Where you born in Chile? 14. Si 🡪 **Go to Module C** 15. No 16. Where are you from? 17. How long do you live in Chile?   Years:  Months: | | | | | | |
| **B1** | **B1 Which?** | **B2** |  | **B3** | **B4** |  | **B5**  **55** | **B5 Which?** | **B6** | **B6 which** | **B7** | **B8** | **B9** |
|  |  |  |  |  |  |  |  |  |  |  |  |  |  |

| **C. Health (The person selected or interviewed responds as representative of the minor selected)** | | | | | | | | | | | | | |
| --- | --- | --- | --- | --- | --- | --- | --- | --- | --- | --- | --- | --- | --- |
| 1. Do you currently smoke? 2. Yes, **three** or more cigarettes per/day 3. Yes, one or two cigarettes per day 4. Yes, occasionally (a few cigarettes a week) 5. No, I stop smoking 6. No, I have never smoked (almost never) 7. In the past month, ¿have you practiced any sport or performed physical activity, for 30 minutes or more? 8. Yes, three or more times per week 9. Yes, once or twice per week 10. Yes, less than four times a month 11. I didn’t practice any sport during the month 12. Not applicable, was unable (for illness, license) 13. In the last year, how often do you drink alcohol? 14. Twice or more per week 15. Between two and four time a month 16. Once a month 17. Ocasionally, only for special events 18. Never | | | | 1. Regarding your weight, do you consider you are: 2. Underweight 3. Normal weight **Go to C6** 4. Overweight 5. Obesity 6. Do not know / No answer 7. Currently, have you been diagnosed overweight or obese by a doctor? 8. Yes 9. No 10. Have you been diagnosed with any chronic disease by a doctor?” 11. Yes 12. No 🡪 **Go to Module D** 13. Which is that disease? | | | | 1. In the last six months, have you received medical treatment due to this disease? 2. Yes 3. No 4. ¿Your disease is covered by the AUGE system?[[5]](#footnote-6) 5. Yes 6. No **🡪 Go to Module D** 7. Do not know **🡪 Go to Module D** 8. Do you use AUGE for the treatment of your disease? 9. Yes 10. No **🡪 Go to Module D** 11. ¿Are you complementing AUGE with a complementary insurance or pay out of pocket? 12. Yes 13. No | | | | | |
| **C1** | **C2** | **C3** |  | **C4** | **C5** | **C6** | **C7** |  | **C8**  **55** | **C9** | **C10** | **C11** |  |
|  |  |  |  |  |  |  |  |  |  |  |  |  |  |

| **D. Medical attention during the last 6 months (The person selected or Interviewed responds as representative of the minor selected)** | | | | | | | | | | | | | | | |
| --- | --- | --- | --- | --- | --- | --- | --- | --- | --- | --- | --- | --- | --- | --- | --- |
| 1. In the last 6 months, Have you needed medical attention (excluding hospitalization)? Multiple answer 2. Yes, I used traditional medicine 🡪**Go to D2** 3. Yes, I used alternative medicine, naturopathic, etc. 4. Yes, I self medicated with pharmacotherapy **Go to D4** 5. Yes, I sought medical attention but I am still waiting 6. No 🡪 Go to **Módule E** | | | | | | 1. What type of medical attention did you use? and, 2. How many consultations you made*? Do not include hospitalization* 3. Preventive care 4. General medicine 5. Specialty medicine 6. Emergency medicine 7. Chronic care 8. Rehabilitative care 9. Palliative care 10. Other. Specify | | | | | | | | | |
| **D1_1** | **D1_2** | **D1_3** | **D1_4** | **D1_5** |  | **D2_1** | **D2_2** | **D2_3** | **D2_4** | **D2_5** | **D2_6** | **D2_7** | **D2_8** | **Specify** |  |
|  |  |  |  |  |  |  |  |  |  |  |  |  |  |  |  |
|  |  |  |  |  |  | **D3_1** | **D3_2** | **D3_3** | **D3_4** | **D3_5** | **D3_6** | **D3_7** | **D3_8** | **Specify** |  |
|  |  |  |  |  |  |  |  |  |  |  |  |  |  |  |  |

| 1. Did you need other type of medical care that you could not receive? *Do not include hospitalization* 2. Preventive care 3. General medicine 4. Specialty medicine 5. Emergency medicine 6. Chronic care 7. Rehabilitative care 8. Palliative care 9. Other. Specify 10. No 🡪 **Go to** **Módule E if did not answer D2 and D3** | 1. What was the reason you could not receive medical attention? Multiple answer  | 1 | 2 | 3 | 4 | 5 | 6 | 7 | 8 | 9 | 10 | 11 | | --- | --- | --- | --- | --- | --- | --- | --- | --- | --- | --- | |  |  |  |  |  |  |  |  |  |  |  | |  |  |  |  |  |  |  |  |  |  |  | |  |  |  |  |  |  |  |  |  |  |  | |  |  |  |  |  |  |  |  |  |  |  | |  |  |  |  |  |  |  |  |  |  |  | |  |  |  |  |  |  |  |  |  |  |  | |  |  |  |  |  |  |  |  |  |  |  |   1. Could not contact a doctor or a medical appointment 8. I do not like doctors  2. Long waiting time 9. I google my sympthoms  3. The service was not available where I live 10. I decide not to seek care  4. Transportation problems 11. Other, Specify  5. Language problems  6. Did not know where to go (information problems)  7. I was very busy |  |
| --- | --- | --- | --- | --- | --- | --- | --- | --- | --- | --- | --- | --- | --- | --- | --- | --- | --- | --- | --- | --- | --- | --- | --- | --- | --- | --- | --- | --- | --- | --- | --- | --- | --- | --- | --- | --- | --- | --- | --- | --- | --- | --- | --- | --- | --- | --- | --- | --- | --- | --- | --- | --- | --- | --- | --- | --- | --- | --- | --- | --- | --- | --- | --- | --- | --- | --- | --- | --- | --- | --- | --- | --- | --- | --- | --- | --- | --- | --- | --- | --- | --- | --- | --- | --- | --- | --- | --- | --- | --- | --- |

| **In general, thinking about medical attentions received in the last 6 months, not considering emergency attention, which of the following options best represents you?** | | | | | | | | | | | | |
| --- | --- | --- | --- | --- | --- | --- | --- | --- | --- | --- | --- | --- |
| 1. From a **monetary** point of view: 2. Medical services, hospital or primary health care center were free 3. Medical services were free, paid by some insurance, firm or other 4. Medical services were paid by FONASA 5. Medical services were paid by ISAPRE 6. Medical services were paid out-of-pocket without any debt 7. Medical services were paid on out-of-pocket with debt 8. I could not afford medical services 9. Regarding the **distance about the place of attention**: 10. I go to the SAPU[[6]](#footnote-7) assigned according to the place where I live 11. I go to the closest public health center (Primary health care center, First Aid Post, Hospital) to my home 12. I go to the closest private health center to my house. 13. I go to the private health center, regardless of its location 14. I visit a private physician regardless of his/her location 15. From the point of view of your need for the attention for **health care specialists:** 16. I received care from a specialist. 17. I received care from a general practitioner 18. I received care from a nurse 19. I received care from other professionals of the health area 20. I received care from other persons not professionals from the health care area 21. I did not received care due to lack of specialists 22. I did not need the attention of a specialist | | | | | | 1. On the need for the attention from the **health care staff:** 2. I received care from a general practitioner 3. I received care from a specialist on alternative medicine 4. I received care from a nurse 5. I received care from other professionals of the health area 6. I received care from other persons not professionals from the health care area 7. I did not received care due to lack of professionals from the health care area 8. I did not need care from the health care staff 9. From a **cultural and/or religious** point of view: 10. I received care without any problem 11. I received care despite the staff did not understand my dialect-language or did not understand my cultural/religious values 12. I did not receive care as the staff could not understand my cultural/religious values 13. I did not receive care as the staff did not understand my dialect/language 14. From a point of view of the **time you regularly wait for** the health service: 15. Very appropriate, I did not wait more than 10 minutes 16. Appropriate, I waited between10 and 30 minutes 17. Reasonably, I waited between 30 minutes and 1 hour 18. Excessive, I waited between 1 and 2 hours 19. Very excessive, I waited more than 2 hours 20. I did not receive care as the waiting time seemed to me extremely excessive | | | | | | |
| **D6** | **D7** | **D8** |  |  |  | **D9** | **D10** | **D11** |  |  |  |  |
|  |  |  |  |  |  |  |  |  |  |  |  |  |

| **Thinking about your last medical attentions received during the past 6 months, which of the following options best represents you?** | | | | | | | | | | | | | |
| --- | --- | --- | --- | --- | --- | --- | --- | --- | --- | --- | --- | --- | --- |
| 1. How did you pay for your medical visit? 2. Medical services, hospital or primary health care center were free **🡪 Go to D16** 3. Medical services were free, paid by some insurance, firm or other **🡪 Go to d16** 4. Medical services were paid by FONASA 5. Medical services were paid by ISAPRE 6. Medical services were paid out-of-pocket without any debt 7. Medical services were paid on out-of-pocket with debt 8. I could not afford medical services **🡪 Go to D16** 9. Did you have to get into debt to pay the value of the consultation? 10. Yes 11. No 🡪 **Go to D15** | | | | | | | 1. How did you cover the health expenses (consultation, treatment or drugs requested) from your visit? 2. A relative/friend 3. FONASA or ISAPRE 4. Department of Welfare from your work 5. Financial institution (bank, credit card or retail) 6. Compensation Fund 7. Other, which? 8. Did you receive reimbursement in your medical visit? 9. Yes, FONASA 10. Yes, ISAPRE 11. Yes, from another health institution (CAPREDENA, DIPRECA, closed ISAPRES) 12. Yes, from the department of welfare from my work 13. Yes, from a private health insurance 14. No | | | | | | |
| **Type of care** | **D12** | **D13** |  |  |  |  | **D14** | **D14 Which?** | **D15** |  |  |  |  |
| Preventive Care |  |  |  |  |  |  |  |  |  |  |  |  |  |
| General Medicine |  |  |  |  |  |  |  |  |  |  |  |  |  |
| Specialty Medicine |  |  |  |  |  |  |  |  |  |  |  |  |  |
| Emergency Care |  |  |  |  |  |  |  |  |  |  |  |  |  |
| Chronic Care |  |  |  |  |  |  |  |  |  |  |  |  |  |
| Rehabilitative Care |  |  |  |  |  |  |  |  |  |  |  |  |  |
| Palliative Care |  |  |  |  |  |  |  |  |  |  |  |  |  |
| Other |  |  |  |  |  |  |  |  |  |  |  |  |  |

| **Thinking about your last medical attentions received during the past 6 months, which of the following options best represents you?** | | | | | | | | | | | | | |
| --- | --- | --- | --- | --- | --- | --- | --- | --- | --- | --- | --- | --- | --- |
| 1. Who was your medical provider? 2. An Specialist doctor 3. A General practitioner 4. A Nurse 5. Other health professional from the health care area 6. Other non-professional from the health care area 7. How was the time allocated for your visit? 8. Very adequate, the attention time was longer than I would have expected 9. Adequate, the specialist dedicated enough time for my attention 10. Moderately adequate, the time was insufficient to solve all my doubts 11. Inadequate, it was not more than 5 minutes 12. Very inadequate, the professional did not show up | | | | | | | 1. How would you evaluate the language used during the visit? 2. Much better than expected, warm, close and using non-medical terms 3. Better than expected, using non-medical terms 4. Good, I understood 5. Worst then expected, I did not fully understand 6. Much worse than expected, distant and full of technicalities 7. What is the distance from your home to the health care provider? 8. I used telemedicine from my home 9. 0 to 2 kilometers 10. 2 to 5 kilometers 11. 5 to 10 kilometers 12. More than 10 kilometers, Specify | | | | | | |
| **Type of care** | **D16** | **D17** |  |  |  |  | **D18** | **D19** | **D19 Which?** |  |  |  |  |
| Preventive Care |  |  |  |  |  |  |  |  |  |  |  |  |  |
| General Medicine |  |  |  |  |  |  |  |  |  |  |  |  |  |
| Specialty Medicine |  |  |  |  |  |  |  |  |  |  |  |  |  |
| Emergency Care |  |  |  |  |  |  |  |  |  |  |  |  |  |
| Chronic Care |  |  |  |  |  |  |  |  |  |  |  |  |  |
| Rehabilitative Care |  |  |  |  |  |  |  |  |  |  |  |  |  |
| Palliative Care |  |  |  |  |  |  |  |  |  |  |  |  |  |
| Other |  |  |  |  |  |  |  |  |  |  |  |  |  |

| **Thinking about your last medical attentions received during the past 6 months, which of the following options best represents you?** | | | | | | | | | | | | | |
| --- | --- | --- | --- | --- | --- | --- | --- | --- | --- | --- | --- | --- | --- |
| 1. How long did you wait for your medical appointment? 2. Less than 1 day 3. Between 1 to 7 days 4. Between 1 to 2 weeks 5. Between 2 weeks to 1 month 6. Between 1 to 3 months 7. More than 3 months 8. Was your visit reschedule to a different date after the initial appointment? 9. Yes, from the medical center reschedule my appointment 10. Yes, I canceled the appointment due to work problems 11. Yes, I canceled due to personal problems 12. Yes, I cancel for other problems 13. Yes, I canceled due to laziness 14. No | | | | | | | 1. How would you rate the following processes:   1. Terrible  2. Bad  3. Regular  4. Good  5. Excellent   1. Requesting an appointment 2. Medical attention 3. Staff attention 4. Considering your experience, would you return to the same medical center? 5. Totally 6. Probably 7. Maybe 8. Probably not 9. Unlikely 10. No 11. Other, Specify 12. Did you have any additional problem when accessing to your medical visit? 13. Yes, Specify 14. No | | | | | | |
| **Type of care** | **D20** | **D21** |  |  |  |  | **D22** | **D23** | **D23 Specify** | **D24** | **D24 Specify** |  |  |
| Preventive Care |  |  |  |  |  |  |  |  |  |  |  |  |  |
| General Medicine |  |  |  |  |  |  |  |  |  |  |  |  |  |
| Specialty Medicine |  |  |  |  |  |  |  |  |  |  |  |  |  |
| Emergency Care |  |  |  |  |  |  |  |  |  |  |  |  |  |
| Chronic Care |  |  |  |  |  |  |  |  |  |  |  |  |  |
| Rehabilitative Care |  |  |  |  |  |  |  |  |  |  |  |  |  |
| Palliative Care |  |  |  |  |  |  |  |  |  |  |  |  |  |
| Other |  |  |  |  |  |  |  |  |  |  |  |  |  |

| **Thinking about your penultimate medical attentions received during the past 6 months, which of the following options best represents you?** | | | | | | | | | | | | | |
| --- | --- | --- | --- | --- | --- | --- | --- | --- | --- | --- | --- | --- | --- |
| 1. How did you pay for your medical visit? 2. Medical services, hospital or primary health care center were free **🡪 Go to D16** 3. Medical services were free, paid by some insurance, firm or other **🡪 Go to d16** 4. Medical services were paid by FONASA 5. Medical services were paid by ISAPRE 6. Medical services were paid out-of-pocket without any debt 7. Medical services were paid on out-of-pocket with debt 8. I could not afford medical services **🡪 Go to D16** 9. Did you have to get into debt to pay the value of the consultation? 10. Yes 11. No 🡪 **Go to D15** | | | | | | | 1. How did you cover the health expenses (consultation, treatment or drugs requested) from your visit? 2. A relative/friend 3. FONASA or ISAPRE 4. Department of Welfare from your work 5. Financial institution (bank, credit card or retail) 6. Compensation Fund 7. Other, which? 8. Did you receive reimbursement in your medical visit? 9. Yes, FONASA 10. Yes, ISAPRE 11. Yes, from another health institution (CAPREDENA, DIPRECA, closed ISAPRES) 12. Yes, from the department of welfare from my work 13. Yes, from a private health insurance 14. No | | | | | | |
| **Type of care** | **D12** | **D13** |  |  |  |  | **D14** | **D14 Which?** | **D15** |  |  |  |  |
| Preventive Care |  |  |  |  |  |  |  |  |  |  |  |  |  |
| General Medicine |  |  |  |  |  |  |  |  |  |  |  |  |  |
| Specialty Medicine |  |  |  |  |  |  |  |  |  |  |  |  |  |
| Emergency Care |  |  |  |  |  |  |  |  |  |  |  |  |  |
| Chronic Care |  |  |  |  |  |  |  |  |  |  |  |  |  |
| Rehabilitative Care |  |  |  |  |  |  |  |  |  |  |  |  |  |
| Palliative Care |  |  |  |  |  |  |  |  |  |  |  |  |  |
| Other |  |  |  |  |  |  |  |  |  |  |  |  |  |

| **Thinking about your penultimate medical attentions received during the past 6 months, which of the following options best represents you?** | | | | | | | | | | | | | |
| --- | --- | --- | --- | --- | --- | --- | --- | --- | --- | --- | --- | --- | --- |
| 1. Who was your medical provider? 2. An Specialist doctor 3. A General practitioner 4. A Nurse 5. Other health professional from the health care area 6. Other non-professional from the health care area 7. How was the time allocated for your visit? 8. Very adequate, the attention time was longer than I would have expected 9. Adequate, the specialist dedicated enough time for my attention 10. Moderately adequate, the time was insufficient to solve all my doubts 11. Inadequate, it was not more than 5 minutes 12. Very inadequate, the professional did not show up | | | | | | | 1. How would you evaluate the language used during the visit? 2. Much better than expected, warm, close and using non-medical terms 3. Better than expected, using non-medical terms 4. Good, I understood 5. Worst than expected, I did not fully understand 6. Much worse than expected, distant and full of technicalities 7. What is the distance from your home to the health care provider? 8. I used telemedicine from my home 9. 0 to 2 kilometers 10. 2 to 5 kilometers 11. 5 to 10 kilometers 12. More than 10 kilometers, Specify | | | | | | |
| **Type of care** | **D16** | **D17** |  |  |  |  | **D18** | **D19** | **D19 Which?** |  |  |  |  |
| Preventive Care |  |  |  |  |  |  |  |  |  |  |  |  |  |
| General Medicine |  |  |  |  |  |  |  |  |  |  |  |  |  |
| Specialty Medicine |  |  |  |  |  |  |  |  |  |  |  |  |  |
| Emergency Care |  |  |  |  |  |  |  |  |  |  |  |  |  |
| Chronic Care |  |  |  |  |  |  |  |  |  |  |  |  |  |
| Rehabilitative Care |  |  |  |  |  |  |  |  |  |  |  |  |  |
| Palliative Care |  |  |  |  |  |  |  |  |  |  |  |  |  |
| Other |  |  |  |  |  |  |  |  |  |  |  |  |  |

| **Thinking about your penultimate medical attentions received during the past 6 months, which of the following options best represents you?** | | | | | | | | | | | | | |
| --- | --- | --- | --- | --- | --- | --- | --- | --- | --- | --- | --- | --- | --- |
| 1. How long did you wait for your medical appointment? 2. Less than 1 day 3. Between 1 to 7 days 4. Between 1 to 2 weeks 5. Between 2 weeks to 1 month 6. Between 1 to 3 months 7. More than 3 months 8. Was your visit reschedule to a different date after the initial appointment? 9. Yes, from the medical center reschedule my appointment 10. Yes, I canceled the appointment due to work problems 11. Yes, I canceled due to personal problems 12. Yes, I cancel for other problems 13. Yes, I canceled due to laziness 14. No | | | | | | | 1. How would you rate the following processes:   1. Terrible  2. Bad  3. Regular  4. Good  5. Excellent   1. Requesting an appointment 2. Medical attention 3. Staff attention 4. Considering your experience, would you return to the same medical center? 5. Totally 6. Probably 7. Maybe 8. Probably not 9. Unlikely 10. No 11. Other, Specify 12. Did you have any additional problem when accessing to your medical visit? 13. Yes, Specify 14. No | | | | | | |
| **Type of care** | **D20** | **D21** |  |  |  |  | **D22** | **D23** | **D23 Specify** | **D24** | **D24 Specify** |  |  |
| Preventive Care |  |  |  |  |  |  |  |  |  |  |  |  |  |
| General Medicine |  |  |  |  |  |  |  |  |  |  |  |  |  |
| Specialty Medicine |  |  |  |  |  |  |  |  |  |  |  |  |  |
| Emergency Care |  |  |  |  |  |  |  |  |  |  |  |  |  |
| Chronic Care |  |  |  |  |  |  |  |  |  |  |  |  |  |
| Rehabilitative Care |  |  |  |  |  |  |  |  |  |  |  |  |  |
| Palliative Care |  |  |  |  |  |  |  |  |  |  |  |  |  |
| Other |  |  |  |  |  |  |  |  |  |  |  |  |  |

| **E. Health Policy (The person selected or Interviewed responds as representative of the minor selected)** | | | | | | | | | | | | | | | | | | | | | | | | | | | | | | | | | | | | | | | | | | | | | | | | | | | | | | | | | | |
| --- | --- | --- | --- | --- | --- | --- | --- | --- | --- | --- | --- | --- | --- | --- | --- | --- | --- | --- | --- | --- | --- | --- | --- | --- | --- | --- | --- | --- | --- | --- | --- | --- | --- | --- | --- | --- | --- | --- | --- | --- | --- | --- | --- | --- | --- | --- | --- | --- | --- | --- | --- | --- | --- | --- | --- | --- | --- | --- |
| Do you know any of the following governmental health programs and its benefits? Use scale of alternatives  **Scale of alternatives**   1. No, I do not know if the programs or its benefits 2. Yes, I know the program but I do not know its benefits 3. Yes, I know the program and partially know its benefits 4. Yes, I know the program and totally know its benefits 5. Yes, I know the program, I know its benefits and I have used it 6. GES (ex AUGE) 7. Cancer National Program 8. Oral Health Program 9. Cardiovascular Health Program 10. Program of sexual and reproductive health 11. Mental Health Program 12. Chile grows with you Program 13. [[7]](#footnote-8)SENDA programs 14. Do you know Other governmental health programs?   Yes  No 🡪 Go to **E11**   1. What is the name of this program? | | | | | | | | | | | | | | | | | | | | 1. How do you rate the affiliation process to your current health insurance? 2. Very easy, as the affiliation to the health insurance of my choice was automatic 3. Easy, as the process of signing the contract with the health insurance of my choice did not have major problem 4. Moderately easy, as the process of signing the contract with the health insurance of my choice presented some difficulties 5. Difficult, because the process of signing the contract with the health insurance of my choice had many difficulties 6. Very difficult, I could not sign the contract with the health insurance of my choice but I did with another insurer 7. I am disaffiliated at the moment 🡪 **Go to module F** 8. According to your criterion, do you consider fair the amount paid to the insurance company? 9. Very fair, the amount I pay is extremely lower in relation to my use of the system 10. Fair, the amount I pay is much lower with regard to the use I make of the system 11. Moderately fair, the amount I pay is lower than what I use the system 12. Unfair, the amount I pay is higher than what I use the system 13. Very unfair, the amount I pay is much higher than what I use the system 14. Extremely unfair, the amount I pay is extremely higher than what I use the system 15. In the last six months, ¿have you considered changing to another health insurance? 16. Yes, I am thinking of changing due to other reason (specify that Reason) 17. Yes, I consider I have not received the promised coverage 18. Yes, I consider the premium is too high 19. Yes, I have received poor quality care 20. Yes, I have been recommended to change 21. No. I am happy with my health care system 22. No. I do not have another option | | | | | | | | | | | | | | | | | | | | | | | | | | | | | | | | | | | | | | |
| **E1** | | **E2** | | | **E3** | | | **E4** | | | **E5** | | **E6** | | **E7** | | | | **E8** | | | **E9** | | | | | | **E10 name of programe** | | | | | | | |  | | | | |  | | **E11** | | | **E12** | | | **E13** | | | |  | | | | |  |
|  | |  | | |  | | |  | | |  | |  | |  | | | |  | | |  | | | | | |  | | | | | | | |  | | | | |  | |  | | |  | | |  | | | |  | |  | | | |
| **F. Characteristics of the Service (The person selected or Interviewed responds as representative of the minor selected)** | | | | | | | | | | | | | | | | | | | | | | | | | | | | | | | | | | | | | | | | | | | | | | | | | | | | | | | | | | |
| 1. What health care facility is available close to your home, regardless if you use it or not? *Multiple answer* 2. General Primary Health Care Center (Municipal or from the National System of Health Services) 3. Rural First Aid Post 4. Health Reference Center or Center for therapeutic diagnosis   1 Available  2 Not available  Do not know   1. [[8]](#footnote-9)COSAM (Center for Community Mental Health) 2. SAPU (Emergency Service for Primary Health Care Center) 3. Post (Emergency Service in Public Hospital) 4. Public Hospital 5. Private Consultation, Medical Center, Clinic or Private Hospital 6. Private Center for Community Mental Health 7. Armed Forces’ Health Facility 8. Medical Organization for Occupational Injury ([[9]](#footnote-10)Mutual de Seguridad in Chile) 9. Other 10. How long is the time of travel from your home to your regular health center or the one you had to go, independent if you use it or not? 11. More than 2 hours 12. Between 1 to 2 hours 13. Between 40 to 60 minutes 14. Between 20 to 40 minutes 15. From 10 to 20 minutes 16. Less than 10 minutes | | | | | | | | | | | | | | | | | | | | | | | | | | | | | | 1. From your home, are there any means of transport that allow to easily and quickly get to the health center you usually use or the one you had to go? Multiple answers 2. Yes, I have a car which makes me get there 3. Yes, there are subway stations that are close to my home which makes me get there 4. Yes, there is a bus network available from my home which makes me get the health center 5. Yes, there are taxis/shared taxis available from my home and I can get there 6. Yes, there are more means of transport from my home so I can get there 7. No, there are no means of transport which prevents me getting to the health center 8. Do you consider business hours at the health center you regularly attend or the one you had to go are reasonable? 9. Yes 10. No, because is not available 24 hours 11. No, because business hours coincide with my religious activities 12. No, because business hours coincide with my political activities 13. No, because business hours coincide with my domestic activities 14. No, because business hours coincide with my work activities 15. Do not know | | | | | | | | | | | | | | | | | | | | | | | | | | | | |
| **F1a** | **F1b** | | | **F1c** | | | **F1d** | | | **F1e** | | **F1f** | | **F1g** | | | **F1h** | | | | **F1i** | | | **F1j** | | **F1k** | | | **F1l** | | |  | | **F3** | | |  | | |  | | | |  | | |  | | | **F2** | | **F4** | | | |  | | |
|  |  | | |  | | |  | | |  | |  | |  | | |  | | | |  | | |  | |  | | |  | | |  | |  | | |  | | |  | | | |  | | |  | | |  | |  | | | |  |  | |
| **F. Characteristics of the Service (The person selected or Interviewed responds as representative of the minor selected)** | | | | | | | | | | | | | | | | | | | | | | | | | | | | | | | | | | | | | | | | | | | | | | | | | | | | | | | | | | |
| The following questions are just for those persons who jumped from question D1, D4 or D5 to Module E   1. On average, how long did you wait to get outpatient care or medical attention? (Remember last medical visit) 2. Less than one day 3. Between 1 day and 7 days 4. Between 1 week and 2 weeks 5. Between 2 weeks and 1 month 6. Between 1 month and 3 months 7. More than 3 months 8. Has never received a medical attention 🡪 **Go to** **F10** 9. How was the time allocated for your visit? (Remember last medical visit) 10. Very adequate, the attention time was longer than I would have expected 11. Adequate, the specialist dedicated enough time for my attention 12. Moderately adequate, the time was insufficient to solve all my doubts 13. Inadequate, it was not more than 5 minutes 14. Very inadequate, the professional did not show up | | | | | | | | | | | | | | | | | | | | | | | 1. How would you evaluate the language used by the physician during the visit? (Remember last medical visit) 2. Much better than expected, warm, close and using non-medical terms 3. Better than expected, using non-medical terms 4. Good, I understood 5. Worst than expected, I did not fully understand 6. Much worse than expected, distant and full of technicalities 7. How would you rate the following processes from your last medical visit: 8. Requesting an appointment   1. Terrible  2. Bad  3. Regular  4. Good  5. Excellent   1. Medical attention 2. Staff attention 3. Considering your experience, would you return to the same medical center? 4. Totally 5. Probably 6. Maybe 7. Probably not 8. Unlikely 9. No | | | | | | | | | | | | | | | | | | | | | | | | | | | | | | | | | | | |
| **F5** | | | **F6** | | |  | | |  | | | |  | | |  | |  | | | | | **F7** | | **F8** | | **F9** | | | |  | |  | |  | | |  |  | | |  | | |  | | |  | | |  | | |  | |  | | |
|  | | |  | | |  | | |  | | | |  | | |  | | | | |  | |  | |  | | | |  | |  | |  | | |  |  | | |  | | |  | | |  | | |  | | |  | |  |  | |

| **F. Characteristics of the Service (The person selected or Interviewed responds as representative of the minor selected)** | | | | | | | | | | | | | | | | | | | | | |
| --- | --- | --- | --- | --- | --- | --- | --- | --- | --- | --- | --- | --- | --- | --- | --- | --- | --- | --- | --- | --- | --- |
| Everyone answers:   1. Were you hospitalized during the last six months? 2. Yes 3. No 🡪 **Go to F13** 4. What was the average time to be assigned a room or bed? 5. Less than one day 6. Between 1 day and 7 days 7. Between 1 week and 2 weeks 8. Between 2 weeks and 1 month 9. Between 1 month and 3 months 10. More than 3 months 11. I was never assigned, why? 12. How was your life affected by waiting for a hospital bed? 13. Added worry, anxiety, stress to me and to my family and friends 14. I could not do my activities in a normal way (dress, drive, etc.) 15. Increased my dependence on family or friends 16. I lost income 17. I lost my job 18. Increased my medicine intake 19. My general health deteriorated; my condition worsened 20. Other, Specify | | | | | | | 1. Did you need surgery during the last 6 months? 2. Yes 3. No 🡪 **Go to F16** 4. What was the average time to received the surgery? 5. Less than one day 6. Between 1 day and 7 days 7. Between 1 week and 2 weeks 8. Between 2 weeks and 1 month 9. Between 1 month and 3 months 10. Between 3 months and 6 months 11. Between 6 months and 12 months 12. More than 1 year 13. I did not receive the surgery, why? 14. How was your life affected by waiting for the surgery? 15. Added worry, anxiety, stress to me and to my family and friends 16. I could not do my activities in a normal way (dress, drive, etc.) 17. Increased my dependence on family or friends 18. I lost income 19. I lost my job 20. Increased my medicine intake 21. My general health deteriorated; my condition worsened 22. Other, Specify | | | | | | | | | | | | | | |
| **F10** | **F11** | **F11 Why?** | **F12** | **F12 Why?** |  |  | **F13** | **F14** | **F14 Why?** | **F15** | **F15 Why?** |  |  |  |  |  |  |  |  |  | |
|  |  |  |  |  |  |  |  |  |  |  |  |  |  |  |  |  |  |  |  |  |

| **F. Characteristics of the Service (The person selected or Interviewed responds as representative of the minor selected)** | | | | | | | | | | | | | | | | | | | | | |
| --- | --- | --- | --- | --- | --- | --- | --- | --- | --- | --- | --- | --- | --- | --- | --- | --- | --- | --- | --- | --- | --- |
| Everyone answers:   1. What is the usual mechanism by which you book an appointment to your health center? 2. Phone 3. In person, at the attention desk 4. On-line reservations Go to **F19** 5. Other 6. Have never received a medical attention 🡪 **End** 7. In your last appointment, how many calls you had to make to get an attention date? 8. How many calls you consider as acceptable? 9. If during your last appointment your specialist gave you a prescription, Did you have access to the medication prescribed? 10. Yes, with no problem 11. No, because I could not afford it 12. No, medication was out of stock 13. No, I did not have time to buy it 14. No, I did not trust the physician 15. No, other reason, Specify | | | | | | | 1. Indicate your level of agreement or disagreement with the following phrases, for reasons to stop attending a health center: 2. Medical attention is too slow   1 Completely agree  2 Agree  3 Neither agree nor disagree  4 Disagree  5 Completely disagree   1. It is too difficult to get there 2. Too expensive 3. Mistreatment by the Staff 4. The staff does not respect my beliefs | | | | | | | | | | | | | | |
| **F16** | **F17** | **F18** | **F19** | **F19 Why?** |  |  | **F20_a** | **F20_b** | **F20_c** | **DF20_d** | **F20_e** |  |  |  |  |  |  |  |  |  | |
|  |  |  |  |  |  |  |  |  |  |  |  |  |  |  |  |  |  |  |  |  |

| **G. Medical expenses (The person selected or Interviewed responds as representative of the minor selected)** | | | | | | | | |
| --- | --- | --- | --- | --- | --- | --- | --- | --- |
| 1. During the last 6 months, have spent in health due to: Multiple Answer 2. Medical attention  | **Expenditure card** | | --- | | | 1. Less than $40 000 | | 2. Between $40.001 and $80.000 | | 3. Between$80.001and$120.000 | | 4. Between $120.001and $160.000 | | 5. Between $160.001 and $200.000 | | 6. Between $200.001and $240.000 | | 7. Between $240.001 and $300.000 | | 8. Between $300.001 and $370.000 | | 9. Between$370.001 and $480.000 | | 10.Between $480.001 and $680.000 | | 11. $680.001 and more | | 12. Do not know |  1. Medications 2. Lab exams 3. Imaging tests 4. Dental services 5. Other professionals and non-medical health services (psychologists, educational psychologists, etc) 6. Glasses or contact lenses 7. Orthesis and/or therapeutic devices 8. Headphones 9. Hospital care (Surgical Ward rights) 10. Home care 11. Other 12. What was the amount of your health expenditures 13. Did you get a reimbursement?   1. yes  2. no  3. do not know   1. What was the amount of the reimbursement for?      |  | **G2** | **G3** | **G4** | | --- | --- | --- | --- | | 1. Medical attention |  |  |  | | 1. Exams and tests |  |  |  | | 1. Homeopathies, medicinal herbs or other alternative medicines |  |  |  | | 1. Medications |  |  |  | | | | | Finally, Do you consider any other barrier to have access to health services?  Comment:  Thank you for your collaboration!  ! | | | | |
| **Item** | **G1** |  | **Item** | | **G2** | **G3** | **G4** |  |
| Medical Consultation |  |  | Medical Consultation | |  |  |  |  |

1. A8 1. Fonasa meaning the Chilean Public Health System managed by the State and sub-classified (A-D) according to socioeconomic income. [↑](#footnote-ref-2)
2. ISAPRE meaning the Chilean Private Health System managed by Private Investors. [↑](#footnote-ref-3)
3. Sistema de FF.AA y de Orden meaning the Armed Forces Health Insurance System. [↑](#footnote-ref-4)
4. SUF, meaning Single Family Subsidy. [↑](#footnote-ref-5)
5. AUGE, a health care reform meaning Universal Access of Explicit Guarantees. [↑](#footnote-ref-6)
6. SAPU, meaning Emergency Primary Care Unit [↑](#footnote-ref-7)
7. SENDA, meaning National Service for the Prevention and Rehabilitation in the Consumption of Drugs and Alcohol. [↑](#footnote-ref-8)
8. COSAM, Center for Community Mental Health [↑](#footnote-ref-9)
9. Mutual de Seguridad, Medical Organization for Occupational Injury [↑](#footnote-ref-10)
